# CAR‐Cell Therapy for Autoimmune Diseases: From the Laboratory to Clinical Practice

**DOI:** 10.1155/jimr/6629562

**Published:** 2026-02-20

**Authors:** Xiao-Peng Zhang, Yi-dong Chen, Qi Yu, Fang Liu, Jia-min Li, Shuang Li, Yi-yu Cheng, Xiao-yu Fu, Qian-xi Xia, Jun-rong Li, Xiao-hua Hou, Liang-ru Zhu

**Affiliations:** ^1^ Department of Gastroenterology, Union Hospital, Tongji Medical College, Huazhong University of Science and Technology, Wuhan, China, tjmu.edu.cn

**Keywords:** autoimmune diseases, cellular immunotherapy, chimeric antigen receptor cells

## Abstract

Autoimmune diseases (AIDs) are characterized by a breakdown in immune tolerance, wherein the patient’s immune system fails to recognize self‐tissues and subsequently attacks the body’s organs and tissues. Although there are many therapeutic medicines targeting the pathogenic mechanisms, there is currently a lack of curative or long‐term symptom control options. Chimeric antigen receptor (CAR) cells are engineered cells that express multifunctional synthetic receptors. These CAR cells specifically target killer pathogenic immune cells and offer a novel approach to treating AIDs by modulating the immune microenvironment. Recent studies have demonstrated satisfactory outcomes with this method in managing autoimmune conditions. In our review paper, we provide a comprehensive summary of previous cases utilizing CAR cell therapy for AIDs. We hope that our review will provide clinicians with more options for the treatment of AIDs.

**Summary**

• Innovative treatment approach: chimeric antigen receptor (CAR) cell therapy represents a significant advancement over traditional methods (including glucocorticoids, immunosuppressants, biologics, and small‐molecules), offering a more targeted way to modulate the immune response in autoimmune diseases (AIDs).
• Analysis of influencing factors: this review synthesizes and analyzes the key factors influencing the efficacy and safety of CAR cell therapy in AIDs.
• Comprehensive clinical summary: this review provides a detailed summary of the application of CAR cell therapy across 12 AIDs, covering both CAR‐T and CAR‐Treg approaches, and discusses their clinical outcomes and adverse events.

## 1. Introduction

**Figure 1:**
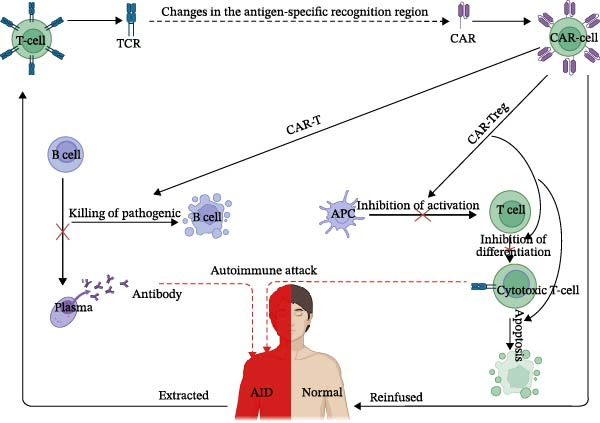
Mechanism of CAR‐cell therapy for autoimmune disease. The process involves extracting T cells from patients with autoimmune diseases (AID) and modifying the specificity antigen recognition region of the T cell receptor (TCR) to create chimeric antigen receptors (CAR). CAR‐T cells are designed to target and eliminate abnormal B cells responsible for producing aberrant plasma antibodies (leading to AID). CAR‐T regulatory (Treg) cells inhibit antigen‐presenting cells (APC) activating T cells to generate cytotoxic T cells (which attack self‐tissues, leading to AID). It also directly suppresses T cells into cytotoxic T cells and induces apoptosis in cytotoxic T cells simultaneously. CAR cells are reinfused into the body to exert their effects in the treatment of AID. (Created in BioRender. Xu, Z. (2025) https://BioRender.com/dnmxzcp). AIDs, autoimmune diseases; APC, antigen‐presenting cells; CAR, chimeric antigen receptors; TCR, T cell receptor.

The concept of chimeric antigen receptors (CARs) was pioneered in 1993 by Zelig Eshhar and colleagues, who used genetic engineering techniques to modify the variable antigen recognition region of T cell receptors (TCR) [1–3]. The native TCR is composed of a variable region that specifically binds to antigens, a hinge region, a transmembrane region, all of which are essential for antigen recognition and the triggering of immune responses. A CAR typically mimics this by combining an intracellular signaling modules, including the cluster of differentiation 3ζ (CD3ζ) chain and one or more costimulatory (CM) domain [4]. This genetic modification enables the generation of CAR T cells from patient’s T cells, endowing them with specific antigen recognition capabilities. Upon infusion of CAR T cell into the host the cells recognize the antigen, become activated, and destroy the target cell (Figure 1) [5].

## 2. Evolution of CAR Generations and Mechanism of CAR‐Cell Therapy

**Figure 2:**
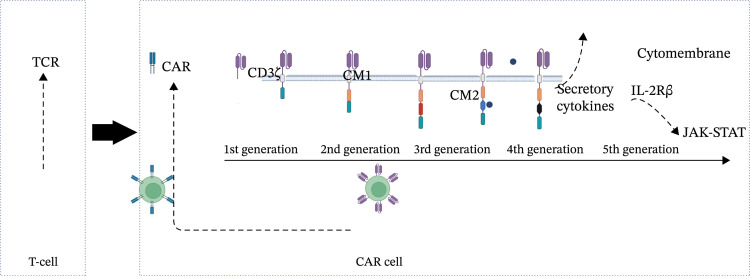
Origin and evolution of CAR generations. T cells extracted from the autoimmune disease (AID) patient. Subsequently, the specificity antigen recognition region of the T cell receptor (TCR) is modified to generate a chimeric antigen receptor (CAR). CAR has developed into the fifth generation. The first generation of CARs contains only CD3ζ; the second generation adds one costimulatory (CM) domain; the third generation adds two CM molecules; the fourth generation adds secretory factors; and the fifth generation incorporate IL‐2Rβ directly activate JAK‐STAT. (Created in BioRender. Xu, Z. (2025) https://BioRender.com/dnmxzcp). CAR, chimeric antigen receptors; CD, cluster of differentiation; CM, costimulatory domain; IL‐2Rβ, Interleukin‐2 receptor subunit beta; JAK‐STAT, Janus kinase and signal transducer and activator of transcription; TCR, T cell receptor.

Currently, the development of CAR technology has reached the fifth generation (Figure 2) [6, 7]. The first‐generation CAR contained a single CD3ζ chain but lacked CM signaling molecules. Although CAR‐T cells are activated in vitro and show cytotoxic effects, these modified cells displayed low cell proliferation and a short in vivo lifespan. The second‐generation CAR incorporated a CM molecule (CD28 or 4‐1BB [CD137]), which significantly increased their cytotoxicity, proliferative activity, and sustained response due to longer in vivo half‐lives [8]. The CD28 facilitated the differentiation of CAR‐T cells into effector memory T cells, while the 4‐1BB extended the duration of CAR‐T cell activity [9–11]. The third‐generation CAR combines multiple CM factors, a combination of CD28 and 4‐1BB or CD28 and OX40 (CD134), not only prolonging CAR‐T cell activation but also enhancing their targeted killing activity [12–14]. Preclinical studies demonstrated that third‐generation CAR‐T cells exhibited significantly higher proliferative capacity and immune factor secretion compared to second‐generation products [13]. However, clinical trials revealed that the initial clinical response rate did not significantly improve [15]. It is hypothesized that excessive stimulation of CAR‐T cells accelerates their exhaustion and leads to more severe toxic side effects. Consequently, current clinical applications still predominantly utilize second‐generation CAR‐T cells. Given that the presence of multiple CM failed to improve CAR‐T cell efficacy, fourth‐generation CAR‐T cells focus on gene modification and remodeling the immune microenvironment, incorporating suicide mechanisms and gene sequences for secretory cytokines like programed death‐ligand‐1 (PD‐L1), interleukin‐12 (IL‐12), and IL‐15. For instance, the fourth generation integrates the *Caspase 9* gene, which can induce cell apoptosis when CAR‐T is activated excessively [16, 17]. Fifth‐generation CAR‐T cells are engineered by incorporating the IL‐2 receptor beta chain (IL‐2Rβ) to activate the intracellular Janus kinase and signal transducer and activator of transcription (JAK‐STAT) [18, 19]. Unlike fourth‐generation CAR‐T cells, which enhance antitumor activity by secreting cytokines upon activation, fifth‐generation CARs incorporate cytokine signaling directly into their intracellular domains, allowing for continuous stimulation and improved resistance to exhaustion. This tri‐modal signaling configuration potentiates sustained T cell activation and augments antitumor potency [20, 21].CAR cells can be categorized into three types based on their mechanisms of action: CAR‐T cells, CAR‐T regulatory (CAR‐Treg) cells, and CAR‐macrophage (CAR‐M) cells. CAR‐T cells eliminate target cells through three distinct mechanisms. First, CAR‐T cells can secrete perforin and granzymes; perforin could create pores in the surface of target cells, allowing granzymes to be delivered into the cells and induce the target cells’ apoptosis. Second, CAR‐T cells express TNF ligands on their surface, which can induce apoptosis in target cells. Additionally, CAR‐T cells can induce cell lysis by secreting specific cytokines. In contrast, the mechanism of action of CAR‐Treg cells is to modulate the overactivated T cells to quiet down [22]. CAR‐Treg cells inhibited antigen‐presenting cells (APCs) to activate T cells to generate cytotoxic T cells. Simultaneously, CAR‐Treg cells could prevent the conversion of T cells into cytotoxic T cells and induce apoptosis in cytotoxic T cells [23]. There are currently no reports on the application of CAR‐M in immune‐related diseases [24].

## 3. Current Therapy for Autoimmune Diseases (AIDs)

AIDs are primarily characterized by the breakdown of immune tolerance, which is the immune system’s ability to distinguish between foreign substances and self‐tissues [25]. Although the exact pathogenic factors of AID remain uncertain, it is well‐established that autoantibodies and disease‐related autoreactive lymphocytes play a significant role in the pathogenesis of these diseases [26]. Historically, therapeutic medicines for AID have focused on treating immune system abnormalities through the use of corticosteroids and immunosuppressants. These agents often sufficiently attenuate the autoimmune inflammatory process, but treatment with them needs to be given continuously over the years, or even lifelong. Even when remission is reached, recurrence of disease often happens when immunosuppression is discontinued [27]. In recent years, biologics and small‐molecule drugs have emerged to target specific organs or cells, resulting in fewer side effects. However, a significant proportion of patients exhibit inadequate responses or intolerance to these agents [28–31]. Furthermore, long‐term use of upon agents is associated with low serum immunoglobulin levels, which increases the risk of infections [32]. For AID driven by B cells and autoantibodies, CAR T‐cell therapy represents a novel and potent strategy for achieving deep B‐cell depletion [33]. Recently, significant strides have been made in expanding patient access to CAR T cell therapy for AID. This strategy stands out not only due to its foundation in production of a personalized, genetically modified autologous cell product, but also because it is designed as a one‐time intervention aimed at achieving sustained, drug‐free remission. This paradigm shift holds the potential to transform AID management from chronic symptom control toward potential cure.

## 4. Current AID of CAR‐Cell Therapy

### 4.1. CAR‐T Cell Therapy for AID (Table 1)

In 2017, CAR‐T cells were approved by the United States Food and Drug Administration (FDA) for tumor treatment. This method has dramatically changed the treatment of hematological malignancies [49]. CAR‐T has emerged as a revolutionary form of cancer immunotherapy [49, 50]. The earliest and most extensively studied target for CAR‐T cells is the CD19 antigen on B cells [49]. Given that B cells and autoantibodies are crucial in the pathogenesis of AID, CAR‐T therapy has increasingly been applied to treat various AIDs in recent years [51].

**Table 1:** CAR‐T therapy for AID.

Disease	Author, reference	Year	CAR target	Subjects (species), *n*	Treatment	Adverse reaction
AIHA	Zhang et al.[34]	2025	CD19	Human (2)	Rapid partial remission achieved (Day 13 and 19).	Adverse events included Grade 1 skin induration in Patient 1 and hypogammaglobulinemia in both patients
ANCA vasculitis	Lodka et al. [35]	2024	CD19	Mouse(5–7)	Mice treated with CD19 CAR T cells showed a rapid, and strong depletion of plasmablasts in spleen, bone marrow, and in the kidneys during the 8‐week observation period.	NA
ANCA vasculitis	Minopoulou et al. [36]	2025	CD19	Human (1)	Circulating CD19+ and CD20+ B cells remained undetectable up to the last day of follow‐up (Day 132)	Early mild CRS (fever without hypotension or hypoxia) and neutropenia (resolved with filgrastim)
ASS	Müller et al. [37]	2023	CD19	Human (1)	Myositis disappeared and respiratory symptoms improved	This patient had an early mild CRS (fever) that resolved after 3 days of treatment with antiinflammatory drugs and biologics
ASS	Pecher et al.[38]	2023	CD19	Human (1)	Muscle pain and fatigue improved rapidly	This patient developed mild CRS in the first week of treatment
MG	Granit et al. [39]	2023	rCAR‐T	Human (14)	The myasthenia gravis score was still decreased at 9 months of follow‐up	This therapy was not associated with dose‐limiting toxicities, CRS, or ICNAS. Common adverse events were headache, nausea/vomiting, and fever, which resolved within 24 h after infusion
MS	Fischbach et al. [40]	2024	CD19	Human (2)	The patient’s disease symptoms were relieved	One patient developed CRS (elevated body temperature) after infusion, which resolved with hormone therapy
SLE	Kansal et al.[33]	2017	CD19	Mouse (14)	Alopecia and proteinuria were improved, the number of CD19B cells was decreased in bone marrow and spleen, and the survival time of mice was prolonged	NA
SLE	Jin et al. [41]	2019	CD19	Mouse (6)	CD19B cells in spleen and lymph nodes of mice were depleted, and the survival time was prolonged	NA
SLE	Mackensen et al. [42]	2022	CD19	Human (5)	Five patients achieved drug‐free remission at 3 months after treatment, with loss of anti‐dsdna antibodies and a more complete reduction in autoantibodies	No ICANS, and CRS occurred in 3 patients (2 with mild fever that improved after 2 days of anti‐inflammatory drug therapy, and 1 with persistent fever that improved after 3 days of biologic therapy)
SLE	Hagen et al. [43]	2024	CD19	Human (1)	Brain and spinal cord regressed, and cutaneous lupus manifestations improved	Despite multiple inflammatory brain lesions, no ICANS were observed
SPS	Faissner et al. [44]	2024	CD19	Human (1)	CRS (grade 2) and sore throat and cervical lymph node swelling successfully treated	Reduced leg stiffness, drastic improvement in gait, walking speed increase over 100%
SPS	Ayzenberg et al. [45]	2025	CD19	Human (1)	CRS (grade 2) and cervical swelling with enlarged lymph nodes successfully treated with paracetamol and tocilizumab.	Decrease of anxiety, stiffness, and an almost complete disappearance of painful MSAs at the third month after infusion
NMOSD	Qin et al. [46]	2020	CT103A	Human (12)	One patient achieved long‐term remission of clinical symptoms, and all the 12 patients showed good tolerance and safety	NA
PV	Lee et al.[47]	2020	DSG3‐CAART	Mouse (10)	Alopecia, skin erosion, and histologic acantholysis resolved	NA
SSc	Bergmann et al. [48]	2023	CD19	Human (1)	Heart, joint, and skin symptoms improved rapidly	Mild fever developed within 24 h after treatment and resolved with the administration of corticosteroids

Abbreviations: AIHA, autoimmune hemolytic anemia; ANCA, antineutrophil cytoplasmic antibody; ASS, antisynthetase syndrome; CRS, cytokine release syndrome; ICANS, immune effector cell associated neurotoxicity syndrome; MG, myasthenia gravis; MS, multiple sclerosis; MSA, muscle spasm attacks; NA, not available; NMOSD, neuromyelitis optica spectrum disorders; PV, *Pemphigus vulgaris*; SLE, systemic lupus erythematosus; SPS, stiff‐person syndrome; SSc, systemic sclerosis.

#### 4.1.1. CAR‐T Cell Therapy for Autoimmune Hemolytic Anemia (AIHA)

AIHA is a rare, acquired disorder characterized by the premature destruction of autologous red blood cells (RBCs) due to autoantibody‐mediated hemolysis [52]. In 2025, Professor Zhang et al. [34] reported two cases of multirefractory AIHA that relapsed after CD19 CAR‐T cell therapy and then responded swiftly to B cell maturation antigen (BCMA)‐targeted T cell engager therapy. Both patients achieved rapid partial remission by Days 13 and 19, respectively [34]. Adverse events included Grade 1 skin induration in Patient 1 and hypogammaglobulinemia in both patients, which resolved with symptomatic treatment [34]. This report suggests BCMA‐targeted therapy as an option for AIHA refractory to CD19 CAR‐T cells.

#### 4.1.2. CAR‐T Cell Therapy for Antineutrophil Cytoplasmic Antibody (ANCA)‐Associated Vasculitis

ANCA‐associated vasculitis (AAV) comprises a group of life‐threatening AID characterized by loss of B cell tolerance toward neutrophil primary granule proteins, mainly proteinase 3 (PR3) or myeloperoxidase (MPO), and subsequent production of ANCAs [36]. ANCAs play a crucial role in AAV pathogenesis by activating neutrophils and monocytes, which in turn induce endothelial injury and organ damage [53]. A tailored treatment regimen based on B‐cell repopulation monitoring has been shown to improve patient outcomes [54]. In a 2024 preclinical study, Professor Lodka et al. [35] demonstrated that CD19 CAR T cells efficiently depleted B cells and plasmablasts, accelerated MPO‐ANCA decline, and protected mice from necrotizing crescentic glomerulonephritis. Subsequently, Professor Minopoulou et al.[36] reported the first clinical case of a patient with severe, treatment‐refractory PR3‐ANCA + AAV treated with autologous anti‐CD19 CAR‐T cells in 2025. Circulating CD19+ and CD20+ B cells remained undetectable through follow‐up (Day 132) [36]. Despite the current limitation of two published reports, emerging evidence supports CAR‐T cell therapy as a promising strategy for managing refractory ANCA‐AAV, particularly in cases resistant to conventional immunosuppressive regimens.

#### 4.1.3. CAR‐T Cell Therapy for Antisynthetase Syndrome (ASS)

ASS is a subtype of idiopathic inflammatory myopathy defined by specific autoantibodies against aminoacyl‐tRNA synthetases. These antibodies, which include those against histidyl (anti‐Jo‐1), tyrosyl (anti‐PL7), alanine (anti‐PL12), glycine (anti‐EJ), isoleucine (anti‐OJ), asparagine (anti‐KS), phenylalanine (anti‐Zo), and threonine (anti‐HA), serve as both markers and triggers of ASS [55]. ASS’s clinical manifestations include myositis, interstitial pneumonia, arthritis, Raynaud’s phenomenon, fever, and skin symptoms. In 2023, professor Müller et al. [37] successfully used CD19 CAR‐T therapy on a patient with idiopathic inflammatory myopathy presenting with anti‐Jo‐1 antibodies, whose myositis disappeared and respiratory symptoms improved after the treatment. Although the patient experienced a mild fever post‐infusion, which subsided after 3 days of anti‐inflammatory and biological therapy. Similarly, Professor Pecher [38] applied this therapy to a patient with ASS complicated by progressive myositis and interstitial lung disease. The patient’s clinical symptoms improved within 2 weeks post‐infusion, and after 8 months of treatment, the overall score was favorable, with only mild fever occurring within 1 week of infusion [38]. These pioneering cases demonstrate the potential feasibility of CD19 CAR‐T therapy for severe ASS. However, larger studies are required to confirm its efficacy and long‐term safety.

#### 4.1.4. CAR‐T Cell Therapy for Myasthenia Gravis (MG)

MG is an AID characterized by the presence of pathogenic plasma cells [56]. In 2023, professor Granit et al. [39] first used RNA‐modified CAR cells (rCAR‐T) to treat individuals with MG. Following the infusion of rCAR‐T cells, patients exhibited an improvement in symptoms and a reduction in the MG severity score lasting up to 9 months [39]. This study supports the feasibility of rCAR‐T as a novel treatment approach for MG.

#### 4.1.5. CAR‐T Cell Therapy for Multiple Sclerosis (MS)

MS is a chronic inflammatory disease affecting the central nervous system. Aberrant autoimmunity plays an important role in disease etiology. Traditionally, activated abnormal T cells have been implicated as the primary triggers of MS. However, recent studies indicate that B cells and innate immune system cells also play significant roles in disease [57]. In 2024, professor Fischbach et al. [40] used CD19 CAR‐T to treat 2 MS patients. Following the infusion, one patient experienced a mild fever, indicative of cytokine release syndrome (CRS), which was successfully managed with corticosteroid treatment. Importantly, neither patient developed immune effector cell‐associated neurotoxicity syndrome (ICANS). These findings suggest the potential feasibility and safety of CD19 CAR‐T therapy as a treatment option for MS [40].

#### 4.1.6. CAR‐T Cell Therapy for Systemic Lupus Erythematosus (SLE)

SLE is a multisystem AID driven by autoantibodies and immune complexes, which cause inflammation and damage in organs such as the kidneys, skin, joints, and central nervous system [58]. Pathogenic autoantibodies are produced by aberrant B lymphocytes. Given that B lymphocytes’ reactivity to DNA and nuclear antigens antedates clinical manifestation of SLE, B cell blockade represents a rational therapeutic approach [59]. In 2017, Professor Kansal et al. [33] attempted to use first‐generation CD19 CAR‐T cells to treat SLE. Experimental results showed that lupus murine skin, bone marrow, and kidney involvement were reduced, with survival times significantly improved [33]. Building on this, in 2020, Professors Yongmei Han, Linrong Lu used second‐generation to explore CD19 CAR‐T cells Prophylactic Effect in the SLE mouse model. Their findings indicated that pre‐infusion of CD19 CAR‐T cells could deplete CD19 B cells in mice, thereby extending the survival duration of the SLE mice [41]. Further advancements were made in 2022 when Professors Mackensen et al. [42] treated five refractory SLE patients with the second‐generation CD19 CAR‐T cells. Notably, none of the patients developed ICANS. Although three patients experienced fever, belonging to mild CRS, which improved following anti‐inflammatory and biologic treatment [42]. In 2023, Professors Heng Mei and Yu Hu from Tongji Medical College successfully achieved remission in a clinical SLE patient using CD19 CAR‐T cells. In 2024, ProfessorHagen et al. [43] had treated central nervous system SLE with CD19 CAR T cells. These studies provide compelling evidence for the feasibility, tolerability, and efficacy of CD19 CAR‐T cell therapy for SLE [60].

#### 4.1.7. CAR‐T cell therapy for Stiff‐Person Syndrome (SPS)

SPS is a rare AID marked by progressive rigidity and episodic muscle spasm attacks (MSA). In 2024, Professor Faissner et al.[44] reported successful use of anti CD19 CAR‐T cells in severe treatment‐refractory SPS. In 2025, Professor Ayzenberg et al.[45] reported that Anti‐CD19 CAR‐T cell therapy in advanced SPS and concomitant MG. In both cases, treatment‐related adverse events included Grade 2 CRS and lymphadenopathy, which resolved with supportive care. Importantly, both patients achieved significant and meaningful clinical improvement. The paradigm‐shifting outcomes in refractory SPS justify accelerated investigation of CD19‐directed CAR‐T cell therapy for neural autoimmunity.

#### 4.1.8. CAR‐T Cell Therapy for Neuromyelitis Optica Spectrum Disorders (NMOSD)

NMOSD is a severe inflammatory disease of the central nervous system, predominantly mediated by pathogenic autoantibodies against aquaporin‐4 (AQP4) [61]. These antibodies bind to astrocytes, leading to complement activation, inflammation, demyelination, and ultimately axonal damage [62, 63]. In 2020, Professor Wei Wang from the Department of Neurology at Huazhong University of Science and Technology, Tongji Medical College, registered a clinical study to evaluate the safety and tolerability of CT103A cell therapy in patients with recurrent/refractory optic neuritis spectrum disorders (ClinicalTrials.gov identifier: 2000038369) [46]. This experiment included 12 cases of neuromyelitis optica patients. Subsequently, these patients underwent an exploration of CAR‐T therapy. In 2023, Professor Wei Wang revealed that a patient with AQP4 antibody‐positive neuromyelitis optica clinical symptoms achieved long‐term remission following CAR‐T treatment. Furthermore, all patients in the cohort demonstrated good tolerability and safety. The success of this case suggests the potential of CAR‐T therapy for treating neuroimmunological diseases. However, as with other emerging indications, data remain limited to small early‐phase studies, underscoring the critical need for larger, controlled trials to definitively establish efficacy and safety.

#### 4.1.9. CAR‐T Cell Therapy for *Pemphigus Vulgaris* (PV)

PV is a potentially life‐threatening autoimmune blistering disease of the skin and mucous membranes [62]. Its pathogenesis is driven by autoantibodies against desmogleins, which are primarily produced by short‐lived plasmablasts [64, 65]. These autoantibodies disrupt the keratinocyte’s desmosomal connections, leading to acantholysis in the basal spinous layer, severely affecting the skin and mucosal functions [62]. In 2020, Professor Jinmin Lee pioneered the use of desmoglein 3 chimeric autoantibody receptor T cells (DSG3‐CAART) as a treatment for PV. This innovative approach demonstrated significant improvements in PV mouse models, including reduced skin alopecia, erosion, and histological acantholysis [47]. Based on this compelling preclinical data, the FDA granted DSG3‐CAART (Cabaletta Bio) designation as a potential therapeutic drug for PV in February 2020. Nevertheless, further research is necessary to evaluate its safety and efficacy in patients with PV [64].

#### 4.1.10. CAR‐T Cell Therapy for Systemic Sclerosis (SSc)

SSc, also known as scleroderma, is an immune‐mediated rheumatic disease. It is characterized by fibrosis of the skin and internal organs, as well as vascular lesions [66]. In 2023, Professor Bergmann et al. [48] treated a patient with severe refractory SSc using CD19 CAR‐T cells successfully. The patient had previously failed to respond adequately to multiple conventional immunosuppressive therapies, including methotrexate and mycophenolate mofetil. Following CAR T‐cell infusion, rapid improvement was observed in cardiac, articular, and cutaneous manifestations. The treatment was complicated by a brief episode of mild CRS (fever) within the first 24 h, which resolved promptly with corticosteroid management. Regrettably, there are no further reports on the use of CAR‐T therapy for treating SSc.

### 4.2. CAR‐Treg Cell Therapy for AID (Table 2)

Regulatory T (Treg) cells, comprising 1% to 2% of peripheral blood lymphocytes, are essential for maintaining immune homeostasis, and are the primary controllers of self‐tolerance. In 2009, Professors Hombach et al. [70] generated the first human CAR‐Treg cells, which could specifically target and adjust the immune environment by modulating overactivated T cells [22, 71].

**Table 2:** CAR‐Treg therapy for AID.

Disease	Author, reference	Year	CAR target	Subjects (species), *n*	Treatment	Adverse reaction
CD	Cui et al. [67]	2025	IL23R	Cell (NA)	IL23R‐CAR displayed negligible tonic signaling and a strong signal‐to‐noise ratio	NA
GvHD	Noyan et al. [68]	2016	HLA‐A2	Mouse (7)	The rejection of allogeneic targets in mice was completely prevented and the survival rate of mice was improved	NA
T1DM	Imam et al. [69]	2019	GAD65	Mouse(unpublished)	The mice that received the treatment had a decrease in blood glucose within one month	NA

Abbreviations: CD, Crohn’s disease; GAD65, glutamic acid decarboxylase 65; GvHD, graft versus host disease; HLA‐A2, human leukocyte antigen‐A2; T1DM, type 1 diabetes; NA, not available.

#### 4.2.1. CAR‐Treg Cell Therapy for Crohn’s Disease (CD)

CD is a chronic, relapsing‐remitting form of inflammatory bowel disease (IBD) characterized by transmural inflammation that can affect any part of the gastrointestinal tract [72, 73]. Its pathogenesis involves a complex interplay of genetic susceptibility, environmental triggers, gut microbiota dysbiosis, impaired epithelial barrier function, and dysregulated mucosal immune responses [67]. Treg cells are essential for immune tolerance and for controlling pathological inflammation, particularly in mucosal tissues such as the gastrointestinal tract [74]. In 2025, professor Cui [67] used IL23R‐CAR Treg cell to suppress IL23R‐expressing tissue activity. Preclinical studies demonstrated that these IL23R‐CAR Tregs retained a stable regulatory phenotype and potently suppressed target cells in vitro. Their in vivo efficacy and safety profile await validation in animal models of CD before clinical translation can be considered.

#### 4.2.2. CAR‐Treg Cell Therapy for Graft Versus Host Disease (GvHD)

GvHD, whether acute or chronic, is one of the major complications of allogeneic transplantation. Despite significant advancements in supportive care over the past few decades, this complication still leads to considerable morbidity and mortality [75]. Previous studies have shown that the ex vivo expansion of CD4(+) CD25(+) CD127(−) Tregs can prevent both acute and chronic GvHD [76–79]. In 2016, Professor Noyan [68] constructed a second‐generation human leukocyte antigen‐2 (HLA‐A2) CAR and generated a lentiviral vector encoding CARs for HLA‐A2 CAR Treg cells. That could effectively maintain phenotype and suppressive function, specifically preventing xenogeneic GvHD in mouse models. Experimental results demonstrated that this therapy completely blocked the rejection response to allogeneic targets, enhancing the post‐organ transplantation survival rate of mice [68].

#### 4.2.3. CAR‐Treg Cell Therapy for Type 1 Diabetes (T1DM)

T1DM is characterized by elevated blood glucose levels due to an absolute deficiency of insulin. It would lead to hyperglycemia and life‐threatening diabetes complications, primarily affecting children and adolescents [80]. Under normal physiological conditions, insulin‐producing β‐cells are not targeted by self‐reactive T cells. However, due to genetic or environmental factors, insulin‐producing β‐cells undergo autoimmune destruction, resulting in an absolute deficiency of insulin [81]. Consequently, strategies focusing on insulin‐producing β‐cell replacement, reduction of β‐cell damage, and regeneration therapies are the key points for curing T1DM [82]. In 2019, professors Shahnawaz Imam et al. [69] conducted experiments using glutamic acid decarboxylase 65 (GAD65)‐specific CAR‐Treg cells in T1DM mouse models. Their experiments demonstrated a reduction in blood glucose levels within 1 month post‐infusion, confirming the potential feasibility of this therapy for T1DM.

## 5. Adverse Reactions and Limitations of CAR Cell Therapy for AID

Although CAR cell therapy has shown remarkable efficacy in AID, it is also associated with adverse reactions. One of the primary concerns is CRS, an immune‐mediated cytokine storm resulting from the overactivation and proliferation of CAR‐T cells. This overactivation leads to an over‐anti‐target cell response, manifesting clinically as a range of systemic inflammatory symptoms. Clinically, CRS includes various systemic inflammatory symptoms such as fever, hypotension, hypoxia, respiratory failure, acute kidney injury, coagulopathy, and multiple organ dysfunction with high serum cytokine levels [83–86]. Evidence confirms that patient‐derived adrenaline propagates this self‐reinforcing pathological loop[87]. Another significant adverse reaction is immune effector ICANS; the mechanisms are currently uncertain [88]. In the context of CAR‐T therapy, damage to the patient’s blood‐brain barrier has been observed, which permits abnormal cytokines to infiltrate the central nervous system [89]. The clinical manifestations of ICANS vary in severity, ranging from mild tremors to cerebral edema, seizures, and death in rare cases. Additionally, long‐term therapeutic success is critically dependent on CAR T‐cell persistence and functional durability. Clinical evidence indicates that therapeutic efficacy correlates with CAR‐T cell expansion, persistence, and memory phenotype acquisition, establishing the prevention or delay of T cell exhaustion as a critical objective [90–92]. CAR‐T cell exhaustion is a concern, occurring when the patient’s T cells fail to sustain the production of CAR‐T cells, potentially leading to disease recurrence due to the limited persistence of CAR‐T cells [93]. Notably, in contrast to oncology experience, reported cases of severe ICANS have been exceedingly rare in AID patients treated with CAR T cells [42, 94]. Nonetheless, careful neurological monitoring as well as prophylaxis against seizures is recommended [95]. Potential mechanisms for neurological events in AID could include localized inflammation secondary to on‐target B‐cell depletion in the central nervous system [96]. While it remains unclear whether CAR‐Treg cells induce adverse reactions like CRS and ICANS, their exhaustion may similarly hinder their efficacy in immune suppression [22, 97]. The FDA and EMA recommended 15 years of follow‐up and recommend lifelong monitoring for patients undergoing CAR therapy. And given that many patients with AID are adolescents or young adults, decades of monitoring might be required [98, 99]. Furthermore, the complex, multistep manufacturing process for autologous CAR cell products results in high costs, currently estimated between $373,000 and $475,000 USD per treatment, excluding associated hospital expenses [100, 101]. This significant financial burden also limits broader clinical access and application, confining most therapies to clinical trials and high‐income healthcare settings. Scaling manufacturing and reducing costs are imperative for the widespread adoption of CAR therapies in AID.

## 6. Summary and Expectation of CAR Cell Therapy for AID

Currently, CAR cell therapy has fundamentally changed the field of hematological malignancies and is beginning to show therapeutic potential in AIDs [42, 102, 103]. T cells, high intrinsic migratory capability, allow deep depletion of B cells in multiple target tissues effectively, including organs with a tight barrier, such as testicles or the brain, that block entry of numerous drugs and even antibodies typically [104, 105]. Compared to existing pharmacological treatments, CAR‐based therapies are increasingly regarded as promising options for treating AIDs due to their fewer side effects and broad application prospects. Future studies should identify tissue‐specific antigens via multi‐omics profiling to minimize off‐target effects and enhance therapeutic efficacy beyond classical CD19 targeting. To fully realize this potential and broaden applicability, several key challenges must be addressed. First, expanding the repertoire of target antigens beyond pan‐B‐cell markers like CD19 is crucial. Discovery of tissue‐specific or disease‐relevant autoantigens through multi‐omics approaches could enable more precise interventions with reduced off‐target risks. Second, improving the safety profile is imperative. This includes engineering next‐generation CAR constructs with enhanced safety switches and a favorable exhaustion profile. Furthermore, leveraging artificial intelligence and advanced in vitro/in silico models to better predict and model human‐specific toxicities prior to clinical trials could de‐risk development. All in all, the design of next‐generation CAR cells for treating AIDs should focus on enhancing their activity and persistence while reducing fee, toxicity, and adverse reactions. In the future, they will likely be used in more immune‐related diseases [106, 107].

## Author Contributions

Xiao‐Peng Zhang wrote the manuscript and has directly accessed and verified the underlying data reported in the manuscript. Yi‐dong Chen, Qi Yu, Fang Liu, Jia‐min Li, Shuang Li, Yi‐yu Cheng, Xiao‐yu Fu, Qian‐xi Xia, Jun‐rong Li, Xiao‐hua Hou, and Liang‐ru Zhu revised the manuscript.

## Funding

This work was supported by grants from the National Key R&D Program of China (Grants 2018YFC0114600 and 2023YFC2507300) and the National Natural Science Foundation of China (Grants 82170547 and 81873558).

## Disclosure

All authors had full access to all the data in the study and accepted responsibility to submit for publication.

## Conflicts of Interest

The authors declare no conflicts of interest.

## Data Availability

Data sharing is not applicable to this article as no datasets were generated or analyzed during the current study.
